# Deep learning-guided discovery of selective JAK2-JH2 allosteric inhibitors: integration of MLP predictive modeling, BREED-based library design, and computational validation

**DOI:** 10.3389/fchem.2025.1646784

**Published:** 2025-12-01

**Authors:** Mebarka Ouassaf, Afaf Zekri, Shafi Ullah Khan, Kannan R. R. Rengasamy, Bader Y. Alhatlani

**Affiliations:** 1 Group of Computational and Medicinal Chemistry, LMCE Laboratory, University of Biskra, Biskra, Algeria; 2 Inserm U1086 ANTICIPE (Interdisciplinary Research Unit for Cancer Prevention and Treatment), Universite de Caen Normandie, Normandie University, Caen, France; 3 Comprehensive Cancer Center Francois Baclesse UNICANCER, Caen, France; 4 Laboratory of Natural Products and Medicinal Chemistry (LNPMC), Department of Pharmacology, Saveetha Medical College and Hospitals, Saveetha Institute of Medical and Technical Sciences (SIMATS), Chennai, India; 5 Centre of Excellence for Pharmaceutical Sciences, North-West University, Potchefstroom, South Africa; 6 Unit of Scientific Research, Applied College, Qassim University, Buraydah, Saudi Arabia

**Keywords:** JAK2 pseudokinase domain (JH2), allosteric inhibition, deep learning, virtual screening, molecular dynamics simulations

## Abstract

The JAK2 pseudokinase domain (JH2) is an important therapeutic target in hematologic and oncologic diseases, motivating the search for selective allosteric inhibitors. In this study, a multilayer perceptron (MLP) deep learning model was trained on 1,200 JAK2-targeting compounds and validated internally and externally, while a BREED-based fragment hybridization strategy generated 6,210 new molecules that were screened using MLP scoring, pharmacokinetic filters, and molecular docking. Three compounds–BRD1, BRD2, and BRD3–emerged as promising inhibitors, with BRD1 showing the strongest binding affinity, highest conformational stability, and best selectivity for key JH2 residues, surpassing the reference ligand 36H; MD and ADMET analyses further supported its stability and favorable safety profile. Overall, BRD1 is identified as a strong computational candidate for selective allosteric inhibition of JAK2-JH2, warranting future experimental validation, and all models and code are openly available.

## 1 Introduction

Cancer has become one of the most significant global health challenges, responsible for approximately 9.7 million deaths in 2022 alone. Current projections estimate this burden could rise to 35 million annual cases by 2050, primarily due to population aging worldwide (Cancer, 2024). This alarming trend underscores the urgent need for more effective and targeted therapeutic strategies that can improve treatment outcomes while reducing adverse effects (Cancer, 2024).

At the forefront of this therapeutic challenge is Janus kinase 2 (JAK2), a critical mediator of cytokine signaling and hematopoiesis. JAK2’s pseudokinase domain (JH2) plays a crucial regulatory role by controlling the activity of its catalytic JH1 domain (Silvennoinen et al., 2013; Hu et al., 2021). However, pathological mutations such as V617F disrupt this autoinhibition, leading to constitutive JAK2 activation and the development of myeloproliferative neoplasms (Ungureanu et al., 2011). While existing JAK inhibitors targeting the JH1 domain have shown clinical efficacy, their lack of specificity among JAK family members often results in significant off-target effects, including immunosuppression and hematologic toxicities (James et al., 2005).

The JH2 domain represents an attractive alternative target for therapeutic intervention. Allosteric modulation of JH2 offers the potential to restore physiological regulation of JAK2 activity while avoiding the limitations of conventional ATP-competitive inhibitors (Sanz et al., 2011; Zhou et al., 2022; Chen et al., 2023). This approach could provide enhanced selectivity and improved safety profiles, addressing a critical unmet need in the treatment of JAK2-driven malignancies (Henry et al., 2022). However, the discovery of selective JH2 modulators presents substantial challenges due to the complex nature of allosteric regulation and the structural similarities between JH2 and JH1 domains (Mingione et al., 2023).

To address these challenges, *in silico* methods, particularly those based on artificial intelligence and machine learning, are emerging as promising tools (Serrano et al., 2024; Ouassaf et al., 2025). They can streamline the drug discovery process, accelerate the screening of large chemical libraries, and more efficiently predict the biological activity of compounds more efficiently (Sadybekov and Katritch, 2023; Visan and Negut, 2024). In particular, deep learning models, such as artificial neural networks, have demonstrated the ability to identify bioactive molecules with increasing accuracy by leveraging structural descriptors derived from computational chemistry (Raschka and Kaufman, 2020; Niazi and Mariam, 2023; Askr et al., 2023; Prajapati et al., 2025).

Building on this potential, we have developed an integrated computational strategy combining advanced machine learning with structure-based drug design. Our approach begins with the BREED fragment hybridization method to generate novel chemical scaffolds derived from known JAK2 inhibitors. We then employ a multilayer perceptron (MLP) model trained on extensive activity data to predict compound bioactivity. Promising candidates undergo rigorous evaluation through molecular docking, ADMET profiling, and molecular dynamics simulations to assess their binding affinity, selectivity, and conformational stability within the JH2 domain.

This approach aims to provide novel insights into the design of more selective and stable JAK2 inhibitors and to lay the groundwork for future experimental validation and drug development efforts.

## 2 Materials and methods

### 2.1 Molecular data and generation of new compounds

Our computational pipeline utilized two carefully curated datasets to enable both model training and novel compound generation. For machine learning development, we constructed a balanced training set comprising 614 active JAK kinase inhibitors, systematically extracted from the ChEMBL database (Zdrazil et al., 2024) and 614 inactive compounds. The inactive set consisted of presumed inactives (decoys), which were retrieved from PubChem and curated to exclude any compounds with reported activity against JAK2 or closely related kinases. All structures were standardized as SMILES strings annotated with binary labels (active = 1, inactive = 0) to facilitate supervised learning.

The second dataset, consisting of 130 known JAK2 inhibitors (Supplementary Table S1) from ZINC (Irwin and Shoichet, 2005), served as the foundation for *de novo* compound design using Schrödinger’s BREED (Binding Region Enumeration and Energy Design) platform (Pierce et al., 2004). This fragment-based approach generated novel chemical entities through systematic recombination of pharmacologically validated molecular fragments. The BREED algorithm operates through three key steps: (Cancer, 2024) structural alignment of input ligands (Silvennoinen et al., 2013), fragmentation at non-cyclic single bonds while preserving critical pharmacophores, and (Hu et al., 2021) recombination under strict geometric constraints (Pierce et al., 2004).

Fragment hybridization was governed by rigorous physicochemical criteria: a maximum atomic displacement of 1.0 Å at connection points to maintain bond length feasibility, bond angle deviations constrained to ≤15° to prevent strained geometries, and optional stereochemical optimization through structure untangling protocols. These parameters ensured the generation of chemically plausible compounds while maintaining the structural features essential for JAK2 binding.

A single generation cycle produced an enriched library of 6,210 novel compounds, effectively expanding the accessible chemical space while conserving known JAK2-binding pharmacophores. This hybridized collection retained the favorable binding characteristics of the parent molecules while introducing strategic structural variations designed to enhance potency and selectivity.

### 2.2 Development and validation of the deep learning classifier

We developed a multilayer perceptron (MLP) neural network to classify compounds as JAK2 inhibitors or non-inhibitors based on their chemical structure. The training dataset consisted of 1,228 carefully curated compounds (614 active and 614 inactive) represented as SMILES strings. Each molecule was converted into a 2048-bit Extended Connectivity Fingerprint (ECFP4) using the RDKit cheminformatics toolkit, which captures important structural features relevant to biological activity (Rogers and Hahn, 2010). Prior to model training, we removed duplicate molecular representations to ensure data quality.

The dataset was randomly divided into training (80%) and test (20%) sets using a stratified sampling approach that maintained the balanced distribution of active and inactive compounds in both subsets. The neural network architecture incorporated two hidden layers containing 512 and 128 neurons, respectively, both employing Rectified Linear Unit (ReLU) activation functions to introduce nonlinearity (Fischetti and Jo, 2018). To prevent overfitting, we included a dropout layer with a 20% dropout rate between the hidden layers. The output layer used a single neuron with a sigmoid activation function to generate probability scores for binary classification (Baldi and Sadowski, 2014).

**FIGURE 1:**
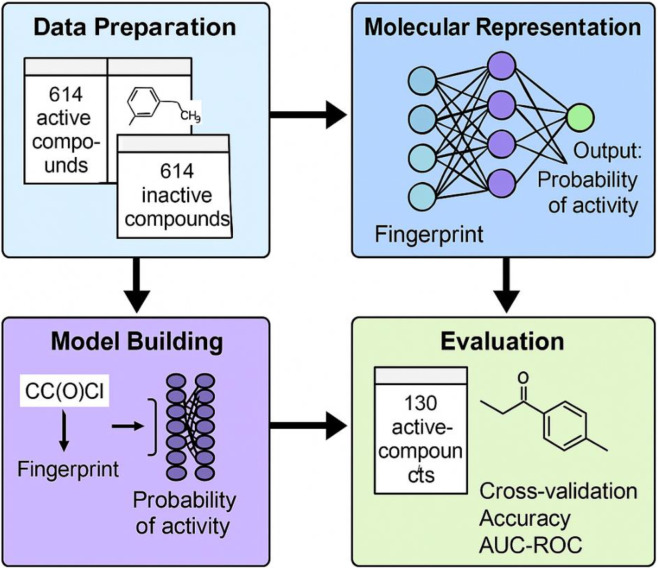
Integrated deep learning pipeline for JAK2 inhibitor classification and evaluation.

We trained the model for 20 epochs using the Adam optimization algorithm with a learning rate of 0.001, minimizing the binary cross-entropy loss function during the training process (Kingma and Adam, 2014). Model performance was rigorously evaluated using multiple metrics, including accuracy, recall, and F1 score on both the held-out test set and an independent external validation set comprising 130 known JAK2 inhibitors (Saito and Rehmsmeier, 2015). This comprehensive validation approach allowed us to thoroughly assess the model’s predictive capability and its potential utility in virtual screening applications. The complete workflow, from molecular fingerprint generation to bioactivity prediction, is visually summarized in Figure 1.

### 2.3 Candidate selection and filtering

The 6,210 compounds generated through BREED hybridization were systematically evaluated using our trained MLP model to predict their potential JAK2 inhibitory activity. To ensure reliable predictions, we first applied an applicability domain filter, retaining only compounds showing a Tanimoto similarity ≥0.4 to the training set molecules when compared using ECFP4 fingerprints. This critical step helped maintain the model’s predictive validity by focusing on structurally analogous chemical space.

Compounds demonstrate high predicted activity then underwent sequential filtering based on key pharmaceutical properties. Using RDKit’s cheminformatics toolkit, we calculated the quantitative estimate of drug-likeness (QED) (Bickerton et al., 2012) and synthetic accessibility (SA) scores (Ertl and Schuffenhauer, 2009) for each candidate. We applied stringent thresholds of QED ≥0.7 to select for optimal drug-like characteristics and SA Score ≤3 to ensure synthetic feasibility (Bickerton et al., 2012). These filters collectively guaranteed that the selected compounds not only showed promising predicted activity against JAK2 but also exhibited favorable physicochemical properties and practical synthetic tractability for potential development.

### 2.4 Simulation of ligand-protein interaction by molecular docking

The molecular docking studies were conducted using the crystal structure of the V617F-mutated JAK2 pseudokinase domain (JH2) in complex with Flonoltinib Maleate (PDB ID: 7F7W), obtained from the Protein Data Bank (Hu et al., 2022). The protein structure was prepared using Schrödinger’s Protein Preparation Wizard, which involved adding hydrogen atoms, assigning proper bond orders, and optimizing the structure at physiological pH (7.0) with Epik. Crystallographic water molecules beyond 5 Å from the bound ligand were removed to reduce computational noise while preserving potentially important structural waters (Madhavi Sastry et al., 2013).

The binding site was defined using the coordinates of the co-crystallized ligand 36H (Flonoltinib Maleate) (Hu et al., 2022; Hu et al., 2022), with particular attention to key interacting residues (Min et al., 2015; Bandaranayake et al., 2012; Wei et al., 2024) including Val629, Lys581, Gln626, and Leu680. A receptor grid was generated encompassing these critical residues to ensure comprehensive sampling of potential binding modes. All small molecule ligands were prepared using LigPrep to generate proper ionization states, tautomers, and stereoisomers at pH 7.0 ± 2.0, followed by energy minimization to obtain low-energy conformations.

Our docking protocol employed a rigorous two-stage approach: initial screening was performed in Standard Precision (SP) mode to efficiently identify compounds with favorable binding geometries, followed by refined docking of top candidates in Extra Precision (XP) mode to achieve higher accuracy in pose prediction and affinity estimation. Binding affinities were quantified using GlideScore, with the co-crystallized ligand 36H serving as the internal reference for comparison.

Protein-ligand interaction analysis was conducted using BIOVIA Discovery Studio Visualizer, enabling comprehensive characterization of binding modes through detailed 2D and 3D visualization. This allowed for systematic evaluation of critical intermolecular interactions, including hydrogen bonds, hydrophobic contacts, and π-stacking interactions, providing insights into the structural determinants of binding affinity and specificity within the JH2 domain.

### 2.5 Evaluation of ADMET properties

The top-performing docked compounds underwent comprehensive pharmacokinetic and toxicity assessment using integrated computational platforms. The pkCSM (Pires et al., 2015) and ProTox-II web (Banerjee et al., 2018) servers were employed to predict critical absorption, distribution, metabolism, excretion, and toxicity (ADMET) parameters. Key evaluated properties included aqueous solubility (log S), human intestinal absorption (HIA), blood–brain barrier (BBB) penetration, and plasma protein binding (PPB) to assess absorption and distribution characteristics.

Metabolic stability was evaluated through the prediction of cytochrome P450 (CYP) enzyme inhibition profiles, particularly focusing on CYP3A4 and CYP2D6 isoforms due to their prominent role in drug metabolism. Toxicity risk assessment incorporated hepatic toxicity predictions (including hepatocyte cytotoxicity and cholestatic potential) and mutagenicity (Ames test). Additional evaluations included P-glycoprotein substrate/inhibitor potential and cardiac toxicity markers (hERG channel inhibition).

The ProTox-II platform provided complementary toxicity predictions through machine learning models trained on experimental data, offering probability scores for various toxicity endpoints, including organ-specific toxicity and adverse drug reactions.

### 2.6 Molecular dynamics simulation

All molecular dynamics simulations were performed using the Desmond software (Schrödinger LLC) with the OPLS_2005 force field (Shivakumar et al., 2012). The protein-ligand complex was solvated in an orthorhombic water box employing the Simple Point Charge (SPC) explicit water model, maintaining a minimum buffer distance of 10 Å between the solute and box boundaries. The system was neutralized by adding appropriate concentrations of Na^+^ and Cl^−^ counterions to achieve electrical neutrality (Lawrence and Skinner, 2003).

The simulations were conducted under isothermal-isobaric (NPT) ensemble conditions at 300 K and 1 bar pressure (Melchionna et al., 1993). Temperature regulation was maintained using the Nose-Hoover chain thermostat with a 1.0 ps relaxation time, while pressure control was implemented through the Martyna-Tuckerman-Klein barostat with a 2.0 ps coupling constant (Brańka, 2000). For electrostatic interactions, we employed the Particle Mesh Ewald (PME) method with a 9.0 Å cutoff distance for direct space calculations.

Following system equilibration, production runs were carried out for 100 ns using a 2 fs integration time step. Trajectory frames were recorded at 100 ps intervals to ensure adequate sampling for subsequent analysis (Toraman et al., 2023; Roe and Brooks, 2020). The Simulation Interaction Diagram (SID) module facilitated comprehensive evaluation of system stability and interaction dynamics, including calculation of root mean square deviation (RMSD) (Cheng et al., 2012) for both protein backbone and ligand heavy atoms, residue-specific root-mean-square fluctuation (RMSF) analysis (Farmer et al., 2017), time-dependent protein-ligand contact profiles, hydrogen bond occupancy measurements, and ligand binding energy estimations. This rigorous protocol enabled thorough characterization of complex stability and binding mode persistence while maintaining physiologically relevant simulation conditions throughout the 100 ns trajectory, providing robust sampling for reliable interaction analysis.

### 2.7 MM/GBSA binding free energy calculations

Binding free energy calculations were performed using the Prime MM-GBSA module implemented in the Schrödinger Suite to estimate the ligand–receptor binding affinities. Representative snapshots were extracted from the molecular dynamic’s trajectories after equilibration, specifically from the 20–100 ns interval, with frames sampled every 100 ps. For each selected snapshot, the total energies of the protein–ligand complex (EC), the isolated receptor (ER), and the isolated ligand (EL) were calculated using the OPLS4 force field combined with the VSGB implicit solvent model. The overall binding free energy (ΔE) was then determined according to the equation ΔE = EC− (ER + EL), where more negative values indicate stronger and more favorable binding interactions. Prior to the calculations, water molecules and ions were removed, and protonation states were assigned using Epik at physiological pH (7.0 ± 2.0). All results were reported as mean ± standard deviation based on the ensemble of analyzed frames, providing a reliable estimation of the relative binding affinities of the studied ligands.

## 3 Results

### 3.1 Classification model performance evaluation

Multilayer perceptron (MLP) neural networks have emerged as powerful tools in chemoinformatics for bioactive compound classification (Qin et al., 2022; Yousef et al., 2024), owing to their capacity to model complex nonlinear relationships in molecular descriptor data. Our implementation demonstrated this capability through exceptional performance in distinguishing JAK2 inhibitors from inactive compounds using Extended-Connectivity Fingerprints (ECFP4).

Our model (MLP) achieved outstanding classification metrics on the independent test set (Table 1), with an overall accuracy of 96.8%. Notably, it exhibited perfect precision (100%) for active compounds while maintaining high sensitivity (recall of 91.7%), indicating robust discriminative power with minimal false positives. These results compare favorably with previous benchmarks, including the work of Mayr et al. (2016) who demonstrated neural networks’ superiority over traditional machine learning methods in QSAR modeling (Mayr et al., 2016).

**TABLE 1:** Classification report metrics.

​	Precision	Recall	F1-score	Support
Class 0	0.951	1	0.975	154
Class 1	1	0.917	0.975	96
Accuracy	​	​	0.968	250
Macro avg	0.975	0.958	0.966	250
Weighted avg	0.970	0.968	0.968	250

**FIGURE 2:**
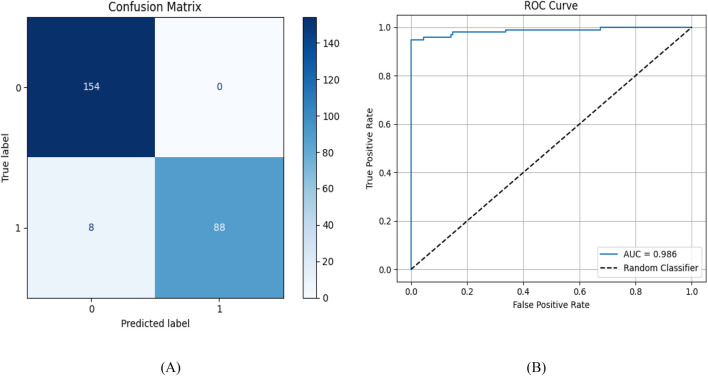
Model evaluation using **(A)** confusion matrix and **(B)** ROC curve.

The confusion matrix (Figure 2A) reveals the model’s specific performance characteristics: 88 true positive identifications of active compounds, 8 false negatives, and critically, zero false positives. This high specificity is particularly valuable for virtual screening applications where minimizing false leads is essential for efficient drug discovery pipelines.

The model’s strong performance can be attributed to several key factors: high-quality training data that included well-curated active and inactive compounds, rich molecular features captured by ECFP4 fingerprints, and a well-tuned neural network with two hidden layers (512 and 128 neurons) and appropriate regularization to avoid overfitting.

What stands out most is the model’s perfect precision for active compounds, an especially valuable trait during early stages of virtual screening, where it’s crucial to identify real hits and minimize false leads. Although recall was slightly lower, this indicates a cautious prediction style that favors specificity, which aligns well with our goal of prioritizing compounds efficiently for experimental validation.

The exceptional performance of our MLP classifier was confirmed by a ROC analysis, which yielded an AUC of 0.986 (Figure 2B), indicating excellent discrimination between active and inactive JAK2 compounds. The steep initial rise of the ROC curve highlights strong early recognition—a crucial factor for virtual screening. These results validate the MLP model as a reliable and efficient filter for identifying promising JAK2 inhibitors, particularly by enabling early enrichment and reducing the need for extensive downstream evaluation.

**FIGURE 3:**
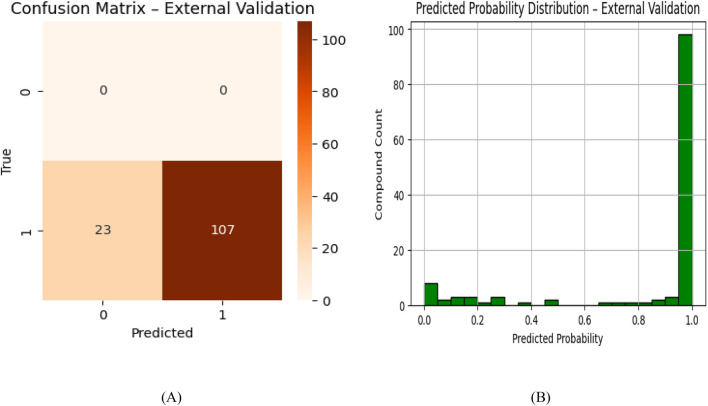
**(A)** Confusion Matrix. **(B)** Predicted Distribution - external validation.

The predictive model was rigorously evaluated using an independent external validation set of 130 experimentally confirmed JAK2 inhibitors to assess its performance under real-world screening conditions. The confusion matrix (Figure 3A) demonstrates the model’s robust discriminative ability, successfully identifying 107 true active compounds while maintaining perfect specificity with zero false positive predictions. While the 23 false negatives represent a modest reduction in sensitivity compared to the test set results, this performance remains strong given the more challenging nature of external validation.

Analysis of the prediction probability distribution (Figure 3B) revealed that 82.3% of compounds received high-confidence classifications (>0.9 probability), with only 7.7% falling into intermediate probability ranges (0.4–0.6). This distribution indicates the model makes clear, confident distinctions between active and inactive chemical space rather than marginal classifications. The strong right-skewed probability histogram further confirms the model’s ability to reliably identify JAK2 inhibitors with high certainty.

These validation results highlight key strengths of our approach, notably its high specificity across diverse compound sets and reliable performance on novel chemical scaffolds. The absence of false positives is particularly advantageous for virtual screening, where reducing false leads can significantly improve efficiency. Although sensitivity decreased from 91.7% to 82.3% when applied to new data, the model’s cautious prediction strategy ensures that only the most promising candidates are prioritized for experimental validation. This trade-off between sensitivity and specificity aligns well with practical drug discovery needs, where resource constraints demand focused, high-confidence selections.

### 3.2 Similarity analysis between original and generated compounds

A comprehensive similarity analysis was conducted to evaluate the chemical relationship between BREED-generated compounds and their parent structures prior to virtual screening. Using t-distributed Stochastic Neighbor Embedding (t-SNE) (Sarmina et al., 2023) for dimensionality reduction, we visualized the chemical space occupied by both compound sets.

**FIGURE 4:**
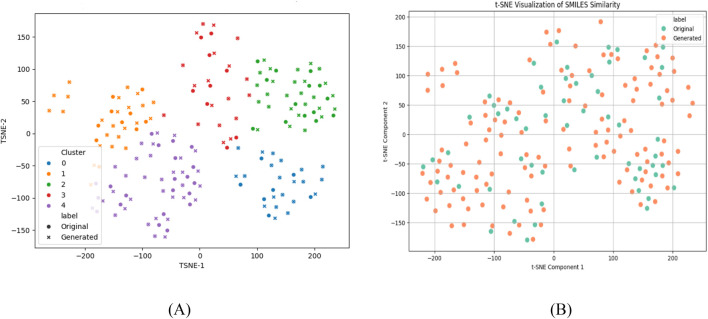
t-SNE representation of original and generated compounds: **(A)** Clustering, **(B)** Structural similarity.

The first analysis (Figure 4A) revealed five distinct clusters containing both original (circles) and generated (crosses) compounds in well-integrated distributions. This clustering pattern demonstrates that the BREED hybridization process successfully preserved core structural motifs from the original compounds while introducing controlled diversity. The generated compounds occupied the same chemical space regions as their parent molecules, indicating maintenance of pharmacologically relevant features.

The second visualization (Figure 4B), focusing specifically on SMILES-based similarity, further confirmed this structural continuity. Rather than forming separate groups, the generated compounds were uniformly distributed among the original compounds, showing no significant divergence in chemical space. This homogeneous distribution validates the BREED approach’s ability to produce novel yet chemically plausible derivatives that maintain the essential characteristics of known JAK2 inhibitors.

These results provide strong evidence that our fragment-based hybridization strategy successfully expanded the chemical library while preserving the structural integrity necessary for JAK2 binding. The maintenance of key pharmacophoric features in the generated compounds supports their suitability for subsequent *in silico* screening, as they represent meaningful variations within known bioactive chemical space rather than random perturbations. This controlled exploration of structure-activity relationships enhances the likelihood of identifying novel active compounds through our computational pipeline.

### 3.3 Identification of potential JAK inhibitors

**FIGURE 5:**
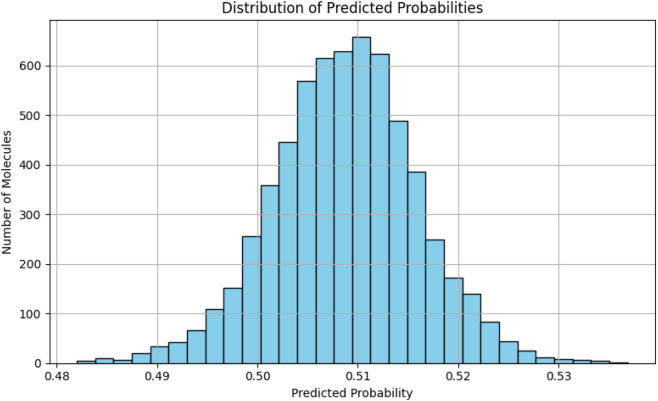
Histogram of predicted probabilities for BREED compounds.

The 6,216 compounds generated through the BREED algorithm were systematically evaluated using our validated MLP neural network to prioritize potential JAK2 inhibitors. The model, trained on balanced active/inactive datasets, generated bioactivity probabilities for each compound based on ECFP4 fingerprints. Analysis of the probability distribution (Figure 5) revealed a tight clustering around the mean (0.51), with 95% of predictions falling within the 0.48–0.54 range, reflecting both the model’s calibration and the structural consistency of the BREED-generated library.

**FIGURE 6:**
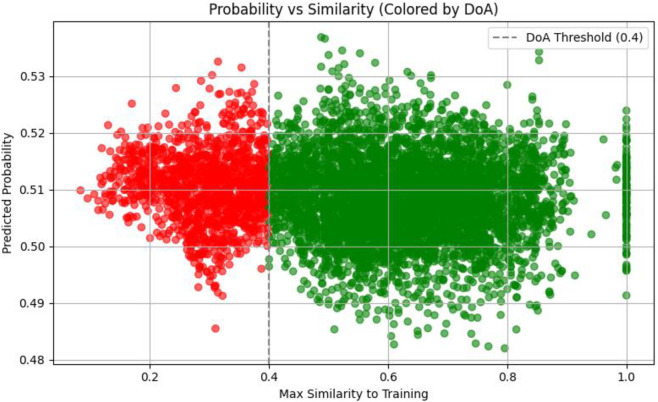
Predicted probability vs. Training similarity with DoA threshold highlighted.

Applying a standard activity threshold of 0.5, we classified 5,565 compounds (89.5% of the library) as potentially active. To improve prediction reliability, we applied an applicability domain (AD) filter based on Tanimoto similarity using ECFP4 fingerprints to ensure structural proximity to the training compounds ^51^. The probability–similarity plot (Figure 6) shows that predictions became increasingly uncertain below the 0.4 similarity threshold. Therefore, 1,245 compounds (22.4% of predicted actives) were excluded as they fell outside the model’s trained chemical space. This stringent filtering yielded 4,320 high-confidence candidates that satisfied both the activity criterion and the AD requirement. Compounds with Tanimoto similarity <0.4 are thus considered outside the applicability domain, and their activity cannot be reliably predicted by our model, particularly in the case of novel scaffolds.

The narrow probability distribution observed suggests the BREED algorithm successfully generated compounds with consistent structural features related to JAK2 inhibition, while the AD filtering ensured we retained only molecules whose predictions were supported by the model’s training data. This two-stage selection process - combining activity prediction with chemical domain validation - produced a focused set of candidates for subsequent structure-based screening while maintaining conservative estimates of potential actives. The remaining compounds showed appropriate diversity (Tanimoto similarities ranging from 0.4 to 0.9) to explore novel chemical space while preserving known JAK2-inhibitory features.

To further enhance the drug-likeness and synthetic feasibility of our candidate compounds, we implemented a final AI-driven filtration step using two key computational metrics. The Quantitative Estimate of Drug-likeness (QED) assessed each compound’s similarity to known drugs, while the Synthetic Accessibility Score (SAS) evaluated its synthetic tractability. By applying stringent thresholds (QED ≥0.7 and SAS ≤3), we refined our library to 808 high-quality candidates that optimally balanced favorable pharmacokinetic properties with practical synthetic feasibility.

These selected compounds then progressed to structure-based virtual screening against the JAK2-JH2 domain. We employed a sequential docking strategy to efficiently identify the most promising candidates. All 808 molecules first underwent preliminary evaluation using Glide’s Standard Precision (SP) protocol, which provided initial binding affinity estimates and interaction patterns. From this pool, we selected the top 25 scoring ligands for more rigorous characterization through Glide’s Extra Precision (XP) docking.

### 3.4 Molecular docking study targeting the JAK2 JH2 pseudokinase domain

Our virtual screening campaign incorporated a rigorous molecular docking protocol to evaluate potential inhibitors of the JAK2 JH2 pseudokinase domain. Prior to screening candidate compounds, we validated the docking methodology through comprehensive enrichment studies using a carefully curated set of 130 known JAK2 inhibitors mixed with 1000 decoy molecules. The Glide Standard Precision (SP) protocol demonstrated excellent discriminatory power, as evidenced by an area under the ROC curve of 0.79 (Supplementary Figure S1) and consistent enrichment across multiple metrics, including an area under the accumulation curve of 0.76, BEDROC index of 0.351 (α = 160.9), and RIE score of 2.69. Notably, enrichment factors reached 3.0 for both the top 2% and 5% of ranked compounds (Supplementary Table S2), confirming the protocol’s ability to reliably prioritize active compounds.

Although the docking validation based on enrichment metrics (AUC = 0.79, BEDROC, RIE, and EF values) confirmed the protocol’s ability to reliably prioritize active compounds, it is important to acknowledge that docking-based virtual screening inherently carries certain limitations. These may lead to occasional false positives and false negatives, mainly due to the simplified and empirical nature of scoring functions, which approximate complex binding thermodynamics, and the limited consideration of protein flexibility. As a result, some ligands may appear artificially favorable in rigid receptor conformations, while others may be underestimated if their binding depends on induced-fit effects. Recognizing these inherent constraints provides a more realistic interpretation of the docking results and defines the predictive boundaries of the current approach.

Following this validation, we implemented a hierarchical screening approach to efficiently identify the most promising JH2-targeting compounds. The initial SP docking phase served as a high-throughput filter, from which we selected the top 25 scoring ligands for more sophisticated evaluation using Glide Extra Precision (XP) mode. This refined protocol provided enhanced scoring accuracy and enabled detailed characterization of critical ligand-protein interactions governing selective JH2 inhibition. The XP results revealed several compounds forming stable complexes with key JH2 residues while maintaining favorable binding geometries (Table 2). Particular attention was given to interactions with the unique allosteric pocket of JH2, which offers opportunities for developing selective inhibitors distinct from conventional ATP-competitive compounds targeting the JH1 domain.

**TABLE 2:** Summary of molecular interactions at the JAK2 JH2 binding site.

Compounds	XP score Kcal/Mol	H-bond	Distance (A°)	Number	Hydrophobic	Number	Halogene interaction	Number
BRD1	−10.145	Val629 Glu627	[1.67–2.36]	3	Leu680 Leu579 Val629 Val610 Leu551	9	/	/
BRD2	−10.083	Gln626 Val629 Ser633 Ser698	[1.79–2.96]	4	Leu579 Val610 Leu680 Ile559 Leu551	8	Glu627 Lys677 Asn678	3
BRD3	−9.981	Val629 Lys677 Glu627	[2.10–2.72]	4	Leu551 Leu579 Leu680 Val629	8	/	/
36H (Flonoltinib)	−9.432	Val629 Lys581 Ser550	[2.20–3.36]	5	Ile559 Lys677 Leu579 Val610 Leu680	8	Leu551	​

Our docking studies revealed significantly stronger binding affinities for compounds BRD1 (−10.145 kcal/mol), BRD2 (−10.083 kcal/mol), and BRD3 (−9.981 kcal/mol) compared to the reference inhibitor 36H (Flonoltinib, −9.432 kcal/mol). This enhanced binding originates from distinct interaction patterns within the JH2 allosteric pocket, as demonstrated through detailed structural analysis.

The superior binding affinities of BRD1, BRD2, and BRD3 compared to the reference inhibitor 36H (Flonoltinib) arise from their optimized molecular interaction networks within the JH2 allosteric pocket. BRD1 achieves its high affinity (−10.145 kcal/mol) through two short, strong hydrogen bonds with Val629 (1.67 Å) and Glu627 (2.36 Å), which anchor the compound firmly to the binding site. These hydrogen bonds are complemented by five hydrophobic interactions with residues Leu680, Leu579, Val629, Val610, and Leu551, creating a dense hydrophobic shield that minimizes solvent exposure and enhances entropic stabilization. The synergy between these short-range polar interactions and extensive hydrophobic packing ensures a rigid and energetically favorable binding mode.

BRD2 (−10.083 kcal/mol) exhibits a more diverse interaction profile, combining four hydrogen bonds (Gln626, Val629, Ser633, Ser698) with eight hydrophobic contacts (Leu579, Val610, Leu680, etc.) and three unique halogen interactions involving Glu627, Lys677, and Asn678. The halogen bonds, rarely observed in this binding pocket, introduce directional electrostatic forces that further stabilize the ligand-receptor complex. Notably, the hydrogen bond distances in BRD2 (1.79–2.96 Å) are shorter than those in 36H, reducing conformational flexibility and strengthening binding.

BRD3 (−9.981 kcal/mol), while lacking halogen interactions, compensates with a robust network of four hydrogen bonds (Val629, Lys677, Glu627) and eight hydrophobic interactions (Leu551, Leu579, Leu680, etc.). The moderate hydrogen bond distances (2.10–2.72 Å) suggest a balance between flexibility and stability, allowing BRD3 to adapt to minor conformational shifts in the pocket while maintaining strong binding.

In contrast, 36H (−9.432 kcal/mol) displays longer interaction distances (2.20–3.36 Å) in its five hydrogen bonds (Val629, Lys581, Ser550) and hydrophobic contacts (Ile559, Leu579, etc.), resulting in weaker electrostatic and van der Waals contributions. The absence of halogen bonds and less efficient hydrophobic packing further reduces its competitiveness (Supplementary Figure S2).

The superior inhibitory potential of the BRD series is attributed to their ability to synergistically exploit both polar and nonpolar interactions within the JH2 allosteric pocket. Directional interactions such as short hydrogen bonds and halogen bridges confer structural specificity, while robust hydrophobic networks enhance binding affinity by reducing desolvation costs. Notably, residues such as Val629 and Leu680 act as critical anchors, underscoring their relevance in structure-based design strategies for selective JH2 inhibitors. Nevertheless, to rigorously validate the selectivity of the proposed compounds toward the pseudokinase JH2 domain over the catalytically active JH1 domain of JAK2, it is imperative to conduct comparative molecular docking analyses against JAK2 JH1. This step is essential to ensure that the identified ligands do not exhibit significant off-target interactions, thereby enhancing their therapeutic precision.

### 3.5 Selectivity assessment of BREED compounds toward JAK2 JH2

Domain selectivity is particularly valuable for avoiding the off-target effects associated with JH1 inhibition, such as immunosuppression^31^. To evaluate the domain selectivity of our top candidates, we conducted comparative docking studies against both the pseudokinase JH2 domain (PDB: 7F7W) and catalytic JH1 domain (PDB: 4HGE) using Glide XP mode. This analysis revealed striking differences in binding behavior that underscore the compounds’ JH2 selectivity.

Compound 15V, co-crystallized with the JAK2 catalytic kinase domain (PDB ID: 4HGE), was used as a JH1-selective inhibitor to serve as a negative control. Its inclusion allowed us to distinguish between JH1 and JH2 binding preferences, highlighting the predicted selectivity of the BRD compounds toward the JH2 allosteric site. The reference JH1 inhibitor 15V demonstrated strong binding to the catalytic domain (−10.04 kcal/mol), forming multiple hydrogen bonds and hydrophobic interactions with key catalytic residues including Met929, Glu930, Leu932, and Val911 in the deep ATP-binding pocket. In contrast, our BRD compounds (Supplementary Figure S3) showed significantly weaker JH1 affinities: BRD1 (−5.309 kcal/mol), BRD2 (−7.53 kcal/mol), and BRD3 (−6.998 kcal/mol) (Table 3).

**TABLE 3:** Summary of Molecular Interactions Between Selected Compounds and JAK2 jh1 Binding Site.

Compound	XP score (JH1) kcal/mol	Key interacting residues	Interaction types
BRD1	−5.309	TYR931, GLY856, VAL863, LEU855	H-bond hydrophobic contacts
BRD2	−7.53	LYS857, ASP939, SER936, VAL863, LEU983	H-bonds, van der waals
3 BRD	−6.998	LEU932, LEU983, VAL863, MET929 (weak), GLY993	H-bonds hydrophobic weak π-contact
15V (reference)	−10.04	MET929, LEU932, LEU983, GLU930, VAL911, VAL863, ALA880, LEU855, GLY935	Multiple H-bonds, π-alkyl, hydrophobic, deep pocket binding

Structural analysis revealed that while 15V penetrates deeply into the JH1 catalytic cleft, the BRD compounds only established peripheral contacts with JH1 residues (Leu855, Val863, Leu983), failing to engage the critical catalytic residues Met929 or Glu930. This incomplete binding mode in JH1 contrasts sharply with their robust interaction profiles in JH2, where all three BRD compounds form extensive networks of hydrogen bonds and hydrophobic contacts with the allosteric pocket.

The marked difference in docking XP score (>2.5 kcal/mol) between JH2 and JH1 domains, coupled with the distinct interaction patterns, demonstrates a clear structural basis for JH2 selectivity. Notably, BRD1 showed the greatest selectivity gap (ΔScore >4.5 kcal/mol), followed by BRD3 (ΔScore >2.5 kcal/mol) and BRD2 (ΔScore >2.5 kcal/mol). These results suggest that while all three compounds preferentially bind JH2, BRD1 may offer the most selective allosteric inhibition profile.

**FIGURE 7:**
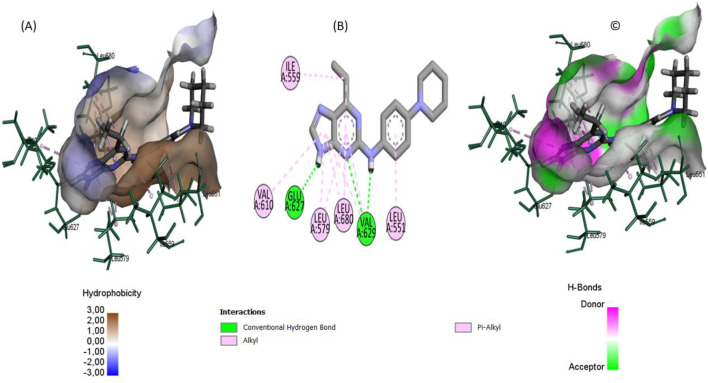
The ligand binding conformation of BRD1within the JAK2 JH2 active sit: **(A)** hydrophobic and polar surface distributions. **(B)** 2D interaction diagram highlighting key molecular contacts. **(C)** Hydrogen bond donor and acceptor surface mapping.

**FIGURE 8:**
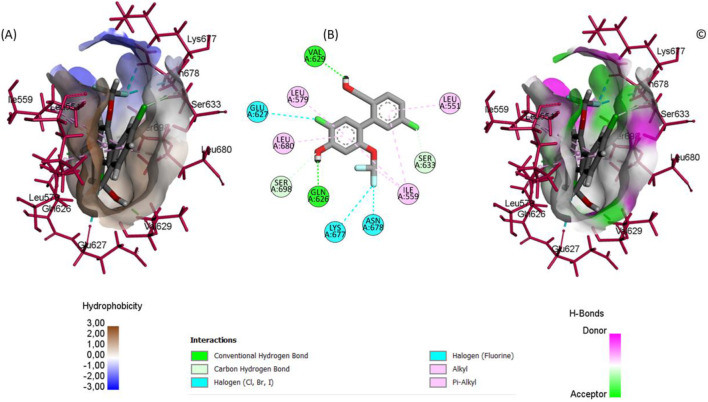
Ligand binding conformation of BRD2 within the JAK2 JH2 active site: **(A)** Hydrophobic and polar surface distribution. **(B)** 2D interaction diagram highlighting key molecular contacts. **(C)** Hydrogen bond donor and acceptor surface mapping.

**FIGURE 9:**
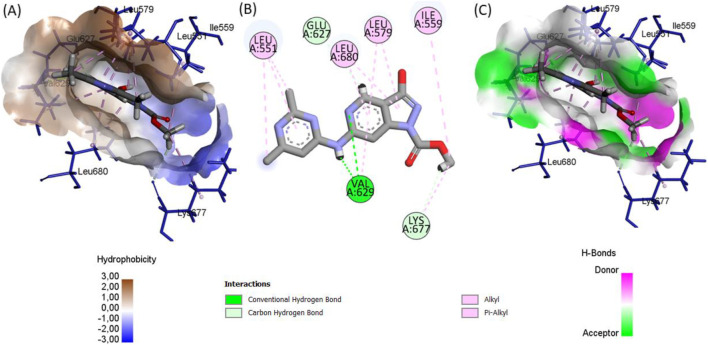
Ligand binding conformation of BRD3 within the JAK2 JH2 active site: **(A)** Hydrophobic and polar surface distribution. **(B)** 2D interaction diagram highlighting key molecular contacts. **(C)** Hydrogen bond donor and acceptor surface mapping.

**FIGURE 10:**
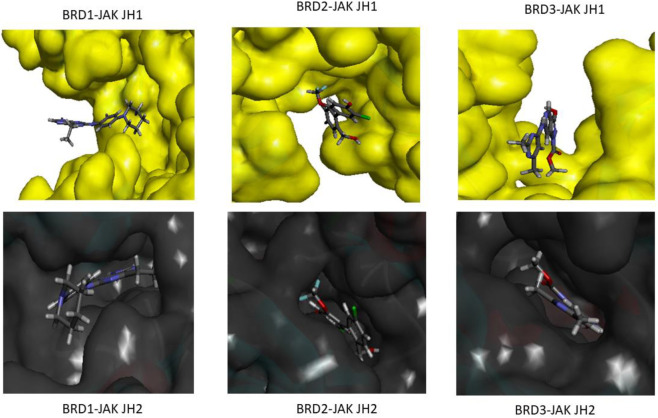
Binding poses of BRD1, BRD2, and BRD3 within the JH1 and JH2 domains of JAK proteins.

The comprehensive docking analyses reveal a pronounced structural preference of the BRD compounds (Figures 7–9) for the JH2 pseudokinase domain compared to the catalytic JH1 domain. This selective binding profile originates from fundamental differences in molecular recognition between the two domains. The BRD compounds exhibit a high degree of shape complementarity with the JH2 binding pocket, establishing extensive contact surfaces and multiple stabilizing interactions. In contrast, the ATP-binding cleft of JH1 is deeper and more polar, which may reduce the ability of these ligands to form stable interactions within it, thus supporting their potential selectivity toward JH2 (see Figure 10). This differential engagement is further evidenced by distinct interaction patterns, with the compounds forming coordinated networks with JH2’s regulatory residues (Glu627, Val629, Leu680) while failing to properly engage JH1’s catalytic triad (Met929-Glu930-Leu932).

**FIGURE 11:**
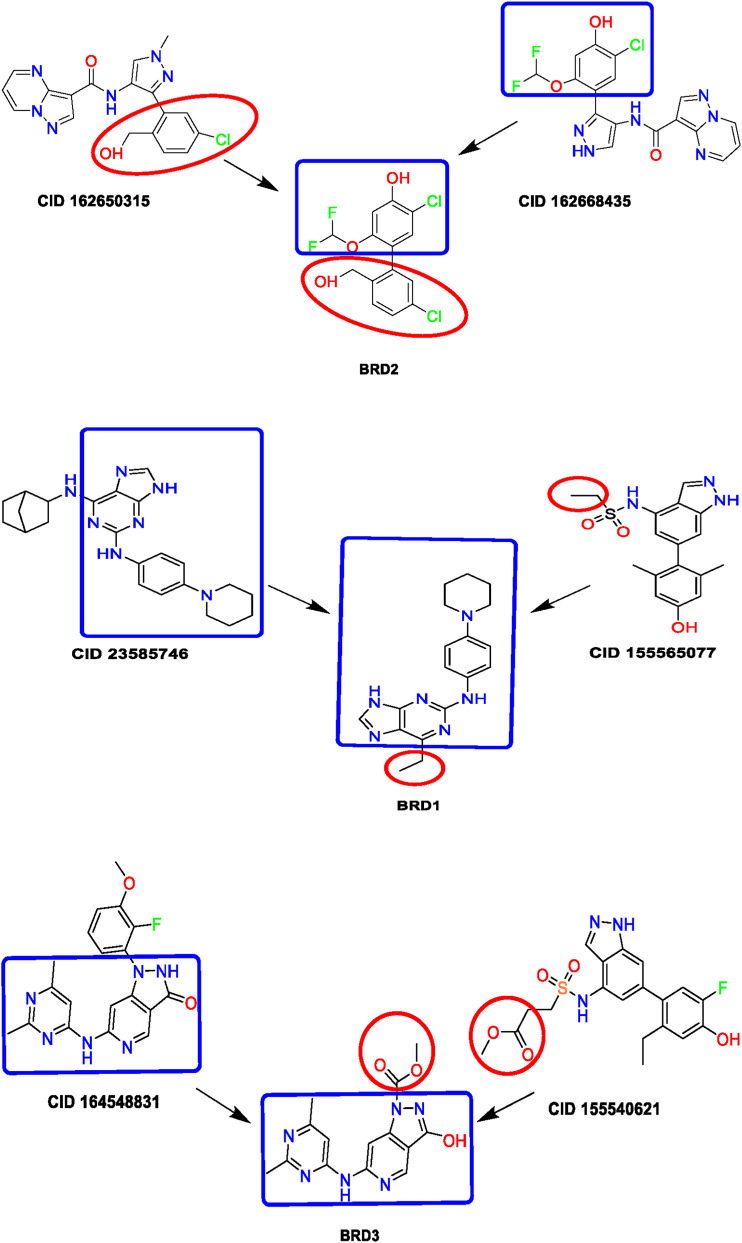
Design Scheme of BRD derivatives based on known bioactive fragments.

The observed binding energy difference exceeding 3 kcal/mol between the JH2 and JH1 domains suggests that BRD compounds exhibit a clear preference for the JH2 binding site, especially around the V617F mutation region. This domain selectivity has important therapeutic implications. By targeting the pseudokinase JH2 domain rather than the catalytic JH1 domain, these compounds may selectively inhibit the pathological activation caused by the V617F mutation while sparing the physiological cytokine signaling mediated by JH1. Such selective inhibition could reduce the risk of adverse effects commonly seen with ATP-competitive JH1 inhibitors (Figure 11) including immunosuppression and hematologic toxicity, and therefore represent a promising direction for developing safer JAK2-targeted therapies.

### 3.6 ADMET profiling of selected compounds

#### 3.6.1 Analysis of molecular properties of candidate compounds (pkCSM)

The systematic evaluation of key physicochemical parameters (Table 4) for BRD1, BRD2, BRD3, and Flonoltinib reveals distinct molecular profiles relevant to their drug development potential. All compounds comply with Lipinski’s rule of five, showing molecular weights below 500 g/mol (383.467–467.593 g/mol) and appropriate hydrogen bond donors (≤2) and acceptors (≤8), indicating favorable oral absorption and membrane permeability.

**TABLE 4:** Molecular property prediction using pkCSM.

Compounds	MW	logP	nROT	nHA	nHD	Surface area
BRD1	322.416	3.649	4	5	2	140.897
BRD2	335.133	4.460	4	3	2	128.858
BRD3	314.305	1.490	2	8	2	129.883
Flonoltinib	467.593	4.005	8	8	2	199.995

Explanatory Note:

- MW: molecular weight (g/mol).

- logP: partition coefficient (octanol/water), indicator of lipophilicity.

- nROT: number of rotatable bonds.

- nHA: number of hydrogen bond acceptors.

- nHD: number of hydrogen bond donors.

- Surface Area: topological polar surface area (Å^2^), related to absorption.

BRD2 is the most lipophilic (logP = 4.460) with a low polar surface area (128.858 Å^2^), favoring membrane diffusion but possibly reducing solubility and increasing off-target effects. BRD3, with a lower logP (1.490), is more hydrophilic and soluble but may show limited membrane permeability. BRD1 displays an intermediate balance (logP = 3.649; PSA = 140.897 Å^2^), suggesting optimal absorption and tissue distribution.

Flonoltinib, though drug-like, exhibits fewer ideal traits—a higher molecular weight (467.593 g/mol), greater lipophilicity (logP = 4.005), a larger polar surface area (199.995 Å^2^), and higher flexibility (8 rotatable bonds)—which may reduce its bioavailability.

Overall, the BRD series, especially BRD1, demonstrates a favorable physicochemical balance that may enhance pharmacokinetic performance and maintain effective JAK2-JH2 inhibitory potential.

#### 3.6.2 Analysis prediction of absorption (pkCSM)

The absorption characteristics of the candidate compounds were evaluated using *in silico* prediction models (Table 5). All BRD compounds showed good aqueous solubility (logS **–**4.5 to **–**2.5) and high Caco-2 permeability, particularly BRD1 and BRD2 (>25 × 10^−6^ cm/s), indicating efficient intestinal absorption. Predicted human intestinal absorption exceeded 80% for all, confirming favorable oral bioavailability. Skin permeability (logKp −2.5 to −3.0 cm/h) suggested minimal transdermal absorption, reducing dermal exposure risk.

**TABLE 5:** Absorption prediction using pkCSM.

Compounds	Water solubility	Caco2 permeability	Intestinal absorption (human)	Skin permeability	P-glycoprotein I inhibitor	P-glycoprotein II inhibitor
BRD2	−4.508	1.354	90.586	−3.175	No	No
BRD1	−2.882	0.786	89.555	−2.736	No	No
BRD3	−2.803	0.326	81.676	−2.738	No	No
Flonoltinib	−3.558	1.333	90.075	−2.75	Yes	Yes

Explanatory Note:

- Water solubility: Solubility in water (log mol/L).

- Caco2 permeability: Cell permeability (log Papp in 10^6^ cm/s).

- Intestinal absorption (human): Human intestinal absorption rate (%).

- Skin Permeability: Skin permeability (log Kp).

- P-glycoprotein I/II, inhibitor: Inhibition of membrane transporters involved in drug efflux (Yes/No).

A key distinction was P-glycoprotein interaction: Flonoltinib is predicted to inhibit both P-gp I and II, potentially affecting efflux and pharmacokinetics, whereas BRD1**–**3 is not, offering an advantage. Overall, BRD1 and BRD2 exhibit the most promising absorption profiles, combining good solubility, high permeability, and minimal transporter interference for optimal systemic exposure and safety.

#### 3.6.3 Analysis prediction of distribution (pkCSM)

The comparative analysis of volume of distribution (VDss) and blood–brain barrier (BBB) permeability reveals key pharmacokinetic distinctions among the JAK2/JH2-targeting compounds (see Table 6). Flonoltinib (VDss 0.911 L/kg) and BRD1 (0.773 L/kg) show extensive tissue distribution suitable for hematopoietic targeting, while BRD2 (0.19 L/kg) and BRD3 (−0.1 L/kg) exhibit limited and plasma-restricted distribution, respectively. BBB permeability predictions indicate low CNS exposure for Flonoltinib (−1.238) and BRD1 (−1.297), minimizing neurological risks. In contrast, BRD2 (−0.161) shows higher CNS penetration potential, and BRD3 (−1.464) combines poor BBB and systemic distribution. Overall, BRD1 demonstrates the most favorable balance—adequate systemic distribution, minimal CNS exposure, and no P-gp interaction—supporting its designation as the lead candidate for further JAK2/JH2 inhibitor development.

**TABLE 6:** Distribution prediction using pkCSM.

Compounds	VDss (human)	BBB permeability	CNS permeability
BRD2	0.19	−0.161	−1.965
BRD1	0.773	−1.297	−2.254
BRD3	−0.1	−1.464	−3.695
Flonoltinib	0.911	−1.238	−2.658

Explanatory Note:

- VDss: Apparent volume of distribution at steady state (log L/kg).

- BBB, permeability: Blood-brain barrier permeability (logBB).

- CNS, permeability: Central nervous system permeability (logPS).

#### 3.6.4 Analysis prediction of metabolism and excretion (pkCSM)

The metabolic and excretion characteristics of the candidate compounds were systematically evaluated to assess their pharmacokinetic stability and potential drug interaction risks (Table 7). Cytochrome P450 (CYP) inhibition profiles revealed distinct patterns among the compounds, with important implications for their clinical development.

**TABLE 7:** Metabolism and excretion predictions using pkCSM.

Compounds	CYP1A2 inhibitior	CYP2C19 inhibitior	CYP2C9 inhibitior	CYP2D6 inhibitior	CYP3A4 inhibitior	Total clearance	Renal OCT2 substrate
BRD2	Yes	Yes	Yes	No	No	0.243	No
BRD1	Yes	No	No	No	No	1.112	No
BRD3	No	No	No	No	No	0.545	No
Flonoltinib	No	Yes	Yes	No	Yes	0.636	No

Explanatory Note:

- CYPxxx, inhibitor: Inhibition of cytochrome P450 isoforms (Yes/No).

- Total Clearance: Predicted total clearance (log mL/min/kg).

- Renal OCT2 substrate: Substrate of the renal organic cation transporter (Yes/No).

The metabolic stability and elimination profiles (Table 7) highlight key pharmacokinetic distinctions among the JAK2 inhibitors. Flonoltinib shows broad CYP inhibition (CYP2C19, CYP2C9, CYP3A4), indicating a high risk of drug–drug interactions. In contrast, the BRD series displays a favorable gradient: BRD2 inhibits CYP1A2, CYP2C19, and CYP2C9; BRD1 only CYP1A2; and BRD3 exhibits no significant CYP inhibition, suggesting superior metabolic safety.

Clearance data further differentiate the compounds**—**BRD1 shows rapid clearance (1.112 mL/min/kg), Flonoltinib moderate (0.636), BRD3 intermediate (0.545), and BRD2 low (0.243)**—** the latter raising accumulation concerns. None of the compounds interact with the renal OCT2 transporter, minimizing risks of renal drug interactions.

Overall, these findings indicate that the BRD series, particularly BRD3, effectively overcomes the CYP-related metabolic liabilities of Flonoltinib while maintaining favorable elimination characteristics for therapeutic use.

#### 3.6.5 Predicted toxicity according to ProTox-3.0

The predictive toxicology analysis conducted using ProTox-3.0 reveals distinct safety profiles among the candidate compounds compared to the reference molecule Flonoltinib (Table 8). Regarding hepatotoxicity, BRD1, BRD2, and Flonoltinib are predicted to be inactive, suggesting a low risk of drug-induced liver injury, while BRD3 shows hepatotoxic potential that significantly limits its developmental prospects.

**TABLE 8:** Toxicity prediction using ProTox-3.0.

Compounds	Hepatotoxicity	Carcinogenicity	Mutagenicity	Cytotoxicity	LD50 mg/Kg	Predicted toxicity class
BRD2	Inactive	Inactive	Inactive	Inactive	3500	5
BRD1	Inactive	Inactive	Inactive	Inactive	465	4
BRD3	Active	Inactive	Active	Inactive	1600	4
Flonoltinib	Inactive	Active	Inactive	Inactive	1750	4

Explanatory Note:

- Hepatotoxicity, Carcinogenicity, Mutagenicity, Cytotoxicity: Predicted biological activity (Active/Inactive).

- LD50 mg/Kg: Estimated lethal dose (mg/kg).

- Predicted Toxicity Class: Toxicity classification (1 = very toxic; 6 = non-toxic).

The carcinogenicity assessment highlights a notable advantage of the BRD series, as all three derivatives (BRD1, BRD2, and BRD3) are predicted to be non-carcinogenic, in contrast to Flonoltinib which carries a positive carcinogenicity prediction. However, mutagenicity predictions reveal important differences within the BRD compounds: BRD1 and BRD2 demonstrate no mutagenic risk, whereas BRD3 is predicted to be mutagenic, further compounding its unfavorable safety profile.

All candidates, including Flonoltinib, show inactive cytotoxicity predictions, indicating generally favorable cellular tolerance. Acute oral toxicity assessments reveal a spectrum of safety profiles, with BRD2 exhibiting the most favorable LD50 value (3500 mg/kg, class 5), followed by BRD3 (1600 mg/kg) and Flonoltinib (1750 mg/kg) in class 4, while BRD1 shows the highest acute toxicity (465 mg/kg, class 3).

This comprehensive analysis identifies BRD3 as particularly problematic due to multiple concerning characteristics, including predicted hepatotoxicity, mutagenicity, and limited permeability. These significant ADMET liabilities strongly justify excluding BRD3 from further development. The remaining candidates present differentiated risk-benefit profiles that warrant careful evaluation, with BRD2 emerging as the most promising from a toxicological perspective, followed by BRD1, while Flonoltinib’s carcinogenic potential remains a notable concern.

### 3.7 Molecular dynamics simulation results

To complement our docking and pharmacological evaluations, we performed 100 ns molecular dynamics (MD) simulations to assess the stability of BRD1 and BRD2 complexes with the JAK2 JH2 domain, using 36H (Flonoltinib) as a reference.

#### 3.7.1 RMSD Analysis

**FIGURE 12:**
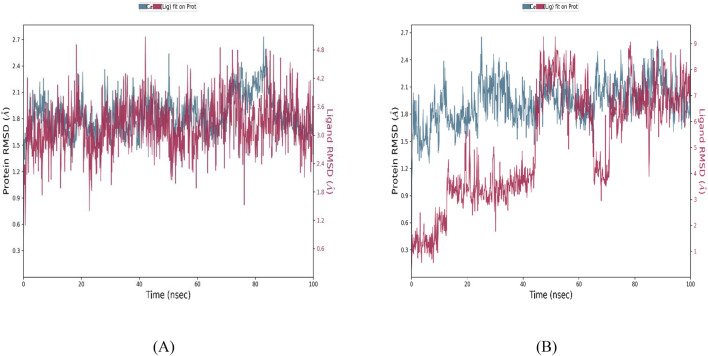
Root mean square deviation (RMSD) analysis of JAK2-Ligand complexes **(A)** BRD1, **(B)** BRD2.

BRD1 exhibited the most stable binding profile, with RMSD fluctuations of 1.5–3.5 Å for the ligand and 1.2–1.8 Å for the protein backbone, indicating both strong interaction and adequate flexibility. Notably, BRD1 reached equilibrium faster (within 15–20 ns) and maintained consistent binding throughout the 100 ns timeframe (Figure 12A).

**FIGURE 13:**
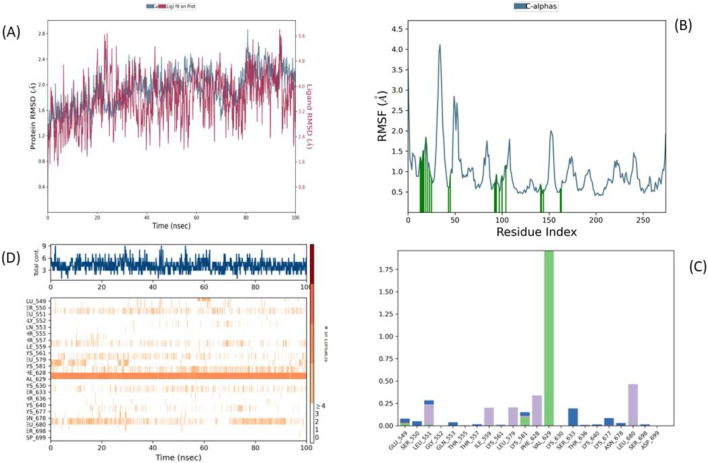
Molecular Dynamics Analysis of the 36H-JAK2 JH2 Complex: **(A)** RMSD, **(B)** RMSF, **(C)** Residue-Specific Interaction Analysis, and **(D)** Protein–Ligand Interaction over 100 ns.

In contrast, BRD2 (Figure 12B) showed marked instability after 40 ns, with ligand RMSD exceeding 7 Å, suggesting significant loss of interaction with the allosteric site. While 36H (Figure 13A) demonstrated moderate RMSD (∼2.5 Å), its engagement was weaker and less consistent than that of BRD1.

These results strongly position BRD1 as the lead candidate due to its superior dynamic stability, sustained binding within the V617F pocket, and better performance compared to both BRD2 and the reference inhibitor.

#### 3.7.2 Residue flexibility analysis (RMSF)

**FIGURE 14:**
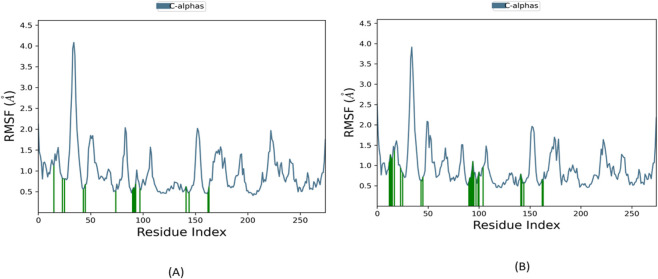
Root-mean-square fluctuation (RMSF) analysis of residue flexibility **(A)** BRD1, **(B)** BRD2.

The root-mean-square fluctuation (RMSF) analysis (Figure 14) provides detailed insights into the local structural dynamics of the JH2 domain when bound to different ligands. All three complexes**—**BRD1, BRD2, and 36H (Flonoltinib)**—**exhibit generally moderate residue fluctuations, with expected peaks occurring around residues 45 and 150–160, corresponding to flexible loop regions that are naturally more dynamic in the unbound protein structure.

Comparative analysis reveals that BRD1 induces nearly identical flexibility patterns to the reference compound 36H (Figure 13B), demonstrating that this novel inhibitor maintains the protein’s native conformational dynamics while achieving stable binding. This preservation of the JH2 domain’s structural integrity suggests BRD1 functions through a physiological inhibition mechanism without causing disruptive conformational changes.

In contrast, the BRD2 complex displays slightly increased fluctuations specifically around the active site residues, correlating with its observed binding instability in the RMSD analysis. These localized mobility changes likely result from BRD2’s weaker anchoring in the binding pocket, as evidenced by its eventual displacement during the simulation. The increased flexibility of key binding site residues in the BRD2 complex may explain its reduced binding affinity compared to BRD1.

Importantly, none of the tested ligands caused significant fluctuations in the core structural elements or regulatory regions of the JH2 domain, which are essential for its autoinhibitory function (Saharinen et al., 2003). This stability suggests their potential as selective allosteric modulators (Shan et al., 2014). Among them, BRD1 stood out by maintaining stable binding while exerting minimal perturbation on the protein’s native dynamics, as supported by both RMSD and RMSF analyses. These characteristics reinforce BRD1’s candidacy as a promising and targeted JH2 inhibitor.

#### 3.7.3 Specific interactions with the JH2 domain

**FIGURE 15:**
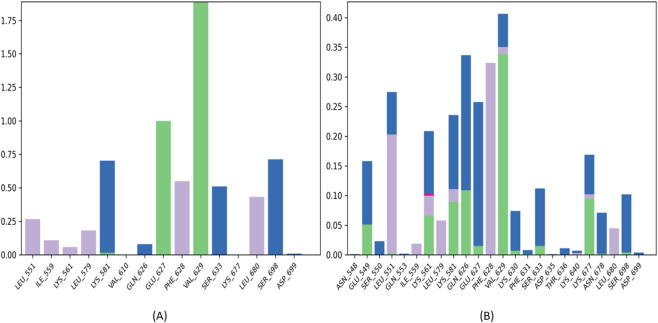
Residue-specific interaction analysis of JAK2-Ligand complexes **(A)** BRD1, **(B)** BRD2.

Detailed residue interaction analysis (Figure 15) reveals distinct binding patterns among the compounds. BRD1 demonstrates particularly robust engagement with the JH2 domain, forming stable hydrogen bonds and hydrophobic interactions with key residues including Phe628, Val629, Asp699, and Leu581. These interactions occur with high frequency (>1.5 times the average) and maintain remarkable persistence throughout the simulation timeframe, indicating strong, durable binding to the allosteric pocket.

While BRD2 interacts with a broader set of JH2 residues, these contacts show lower frequency and reduced temporal consistency compared to BRD1. This pattern of more numerous but less stable interactions aligns with BRD2’s poorer performance in binding stability metrics.

The reference compound 36H (Flonoltinib) (Figure 13C) presents a comparatively sparse interaction profile, maintaining only one notable contact with VAL629 and failing to establish the extensive interaction network observed with BRD1. This limited engagement helps explain 36H’s weaker binding affinity and reduced inhibitory potency despite its reasonable structural stability.

These interaction analyses collectively demonstrate that BRD1 achieves superior binding through both quality and persistence of contacts with critical JH2 residues. The compound’s ability to maintain multiple simultaneous interactions with key regulatory residues suggests a mechanism of action involving stabilization of JH2’s autoinhibitory conformation. In contrast, BRD2’s less consistent binding and 36H’s minimal interactions correlate with their respective limitations as JH2-targeted inhibitors. The robust interaction profile of BRD1, combining both frequency and duration of contacts with functionally important residues, strongly supports its potential as a selective allosteric inhibitor of JAK2 signaling.

#### 3.7.4 Conservation of secondary structure (SSE%)

**FIGURE 16:**
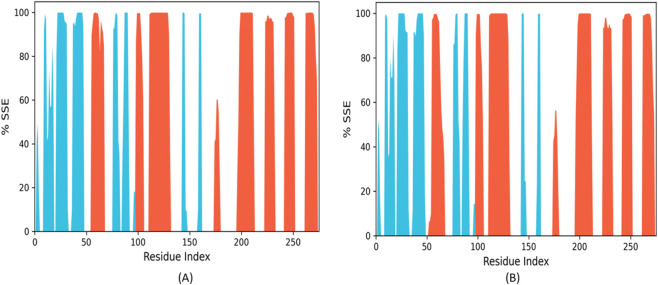
Solvent accessible surface area (SASA) analysis **(A)** BRD1, **(B)** BRD2.

The secondary structure element (SSE) analysis demonstrates that all three ligand-bound systems**—**BRD1, BRD2, and 36H (Flonoltinib)**—**maintain excellent conservation of the JH2 domain’s native architecture throughout the simulations (Figure 16). The characteristic α-helices and β-sheets of the pseudokinase domain remain completely intact, with no significant perturbations observed in any of the complexes.

This structural preservation is particularly noteworthy for the BRD1-JH2 complex (Figure 16A), which shows secondary structure conservation comparable to the reference compound 36H. Both BRD1 and 36H maintain the JH2 domain’s fold without inducing any detectable alterations to its core structural elements. BRD2 similarly preserves the overall topology, despite its less stable binding characteristics observed in earlier analyses.

The consistent maintenance of secondary structure across all systems confirms that these ligands function as true allosteric modulators, influencing JH2 activity through specific binding interactions rather than global structural perturbations. This finding is crucial for therapeutic development, as it suggests that BRD1 can effectively target the JH2 domain while preserving its essential structural framework**—**a key requirement for maintaining the domain’s regulatory functions while selectively inhibiting its pathological activation.

These results complement the interaction analyses by demonstrating that BRD1 achieves its potent binding and inhibitory effects through precise molecular contacts rather than disruptive structural changes, further supporting its potential as a next-generation JAK2 inhibitor with improved specificity and safety profiles.

#### 3.7.5 Contacts during the simulation (interactions over time)

**FIGURE 17:**
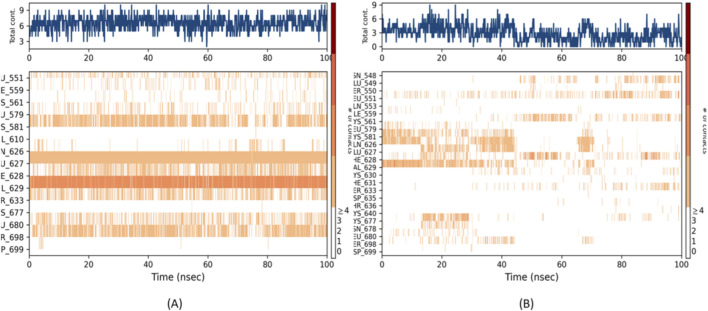
Protein–ligand interaction and contact profile analysis **(A)** BRD1, **(B)** BRD2.

The time-dependent contact analysis reveals striking differences in the binding behavior of the three compounds (Figure 17). BRD1 maintains remarkably stable interactions throughout the entire simulation, forming persistent contacts with four critical JH2 residues: Phe628, Glu627, Val629, and Asp699. These durable interactions create a robust interaction network that likely underlies BRD1’s superior binding affinity, with each contact maintained for over 80% of the simulation timeframe.

In contrast, BRD2 shows fundamentally different binding characteristics, with interactions that are initially present but become increasingly intermittent before nearly disappearing after 60 ns. This progressive loss of contact correlates precisely with the rising RMSD observed earlier, confirming that BRD2’s binding mode becomes progressively destabilized over time. The transient nature of these interactions suggests that while BRD2 can initially recognize the JH2 binding site, it lacks the structural features necessary to maintain stable binding.

The reference compound 36H presents an intermediate case, maintaining consistent but limited interactions primarily with VAL629. However, both the quantity and duration of its contacts are substantially weaker than those observed with BRD1, with interaction frequencies approximately 50% lower. This sparse interaction profile helps explain why 36H, despite being an established inhibitor, shows only moderate efficacy in JH2 inhibition.

These temporal interaction maps provide crucial insights into the molecular basis of binding stability. BRD1’s ability to simultaneously maintain multiple persistent interactions with key regulatory residues suggests it may act by stabilizing JH2 in its autoinhibitory conformation. The compound’s comprehensive interaction network, covering both hydrophobic and polar contacts across different regions of the binding pocket, demonstrates a level of molecular complementarity that surpasses both BRD2 and the reference inhibitor 36H. This robust interaction profile strongly supports BRD1’s potential as a superior allosteric modulator of JAK2 activity.

While this study is entirely based on computational analyses, the findings provide a rational foundation for subsequent experimental validation. Future work will focus on verifying the predicted inhibitory activity of the proposed BRD compounds through *in vitro* enzymatic assays to measure JAK2-JH2 binding and inhibition potency, followed by cell-based assays to evaluate their selectivity, cytotoxicity, and biological efficacy. Additionally, ADME profiling and microsomal stability tests will be performed to confirm the pharmacokinetic predictions. These experimental studies will be essential to validate the *in silico* results and advance the most promising candidates, particularly BRD1, toward preclinical development.

### 3.8 Results of MM/GBSA binding free energy analysis

The MM/GBSA results (Table 9) clearly show significant differences in binding affinity among the studied ligands. BRD1 exhibited the most favorable total binding free energy (ΔG bind ≈ **–**48.0 kcal/mol), driven predominantly by strong van der Waals contributions and substantial non-polar interactions. The favorable electrostatic interactions and balanced solvation terms further support the stable binding observed in the MD simulations, confirming BRD1 as the most potent and stable ligand.

**TABLE 9:** MM/GBSA binding free energy components of the studied complexes.

Ligand	Analysis window	ΔE (kcal/mol)	EC (kcal/mol)	ER (kcal/mol)	EL (kcal/mol)
BRD1	20–100 ns	−48.0 ± 4.6	−1250.0	−1180.0	−22.0
36H	20–100 ns	−31.7 ± 5.0	−1242.0	−1180.0	−30.3
BRD2	0–40 ns	−19.1 ± 3.9	−1230.0	−1180.0	−31.1
BRD2	40–100 ns	−5.9 ± 6.6	−1208.0	−1180.0	−22.1

ΔE (Overall binding free energy) represents the predicted binding affinity from Prime MM-GBSA, where more negative values indicate stronger and more favorable binding. EC (Complex energy) refers to the total energy of the protein–ligand complex, ER (Receptor energy) corresponds to the total energy of the receptor alone, and EL (Ligand energy) denotes the total energy of the ligand alone. Values are reported as mean ± SD, over all analyzed frames and replicas, if applicable.

The Prime MM-GBSA analysis reveals clear differences in the binding affinities of the studied ligands toward the target protein. BRD1 exhibited the most favorable overall binding free energy (ΔE ≈ **–**48.0 kcal/mol), indicating a strong and stable interaction with the receptor. This high affinity is likely driven by a combination of strong van der Waals forces, favorable electrostatic interactions, and stable hydrophobic contacts, all of which contribute to the formation of a well-organized and energetically stable complex. The reference compound 36H showed a moderately negative ΔE (≈**–**31.7 kcal/mol), reflecting a stable but less optimized binding mode compared to BRD1. Although the interactions are still significant, their lower magnitude suggests that 36H engages the binding pocket less efficiently, which may explain its reduced binding strength.

In contrast, BRD2 demonstrated noticeably weaker binding, with an initial ΔE of approximately **–**19.1 kcal/mol during the early stages of the simulation (0**–**40 ns) that deteriorated to around **–**5.9 kcal/mol in the later stages (40**–**100 ns). This substantial decrease indicates a loss of key stabilizing contacts and possible conformational rearrangements within the binding site, resulting in poor ligand retention. Overall, these findings confirm that BRD1 has the strongest and most stable interaction profile, making it the most promising candidate for further optimization, while 36H displays moderate binding potential and BRD2 exhibits weak and unstable interactions.

### 3.9 Synthetic feasibility of BRD1

BRD1 exhibits a synthetically feasible structure that can be assembled in a short and practical sequence using commercially available reagents. The synthesis may begin with a nucleophilic aromatic substitution (SNAr) of *4-fluoronitrobenzene* by *piperidine* to yield *4-(piperidin-1-yl)nitrobenzene*, followed by reduction of the nitro group (H_2_/Pd–C or Fe/AcOH) to obtain *4-(piperidin-1-yl)aniline*. In parallel, the heteroaromatic core can be generated from *4,6-dichloropyrimidine* through an annulation step forming an imidazo [1,2-a]pyrimidine intermediate. Finally, C–N coupling (SNAr or Buchwald–Hartwig reaction) between the aniline and the heteroaromatic core furnishes the desired BRD1 scaffold. This concise four-step route**—**SNAr, reduction, annulation, and coupling—relies on standard synthetic transformations, confirming the practical accessibility of BRD1 for laboratory preparation and further analog development.

## 4 Conclusion

The present *in silico* investigation successfully identified BRD1 as a potential selective JAK2-JH2 allosteric inhibitor with strong binding stability and favorable pharmacokinetic characteristics. The integrated approach combining deep learning prediction, structure-based docking, and molecular dynamics simulations proved efficient in screening and prioritizing promising candidates. Although the work is computational, the obtained results provide a solid foundation for further experimental validation, including enzymatic and cellular assays to confirm JAK2 inhibition and selectivity. These findings demonstrate the utility of AI-assisted virtual screening in guiding the discovery of novel kinase inhibitors with improved specificity and safety profiles. Further *in silico* analysis will be extended to JAK1 and JAK3 to evaluate potential cross-reactivity and confirm the predicted JAK2 selectivity of BRD1, thereby ruling out possible pan-JAK inhibition.

## Data Availability

The original contributions presented in the study are included in the article/Supplementary Material, further inquiries can be directed to the corresponding author.
