# Supplementary material for: Deep learning-guided discovery of selective JAK2-JH2 allosteric inhibitors: integration of MLP predictive modeling, BREED-based library design, and computational validation
Source: Front Chem. 2025 Dec 1;13:1646784. doi: 10.3389/fchem.2025.1646784 (PMC12702926; doi:10.3389/fchem.2025.1646784)
Supplement: Supplementary file 1 [file DataSheet1.docx]

**Deep Learning-Guided Discovery of Selective JAK2-JH2 Allosteric Inhibitors: Integration of MLP Predictive Modeling, BREED-Based Library Design, and Computational Validation**

Mebarka Ouassaf^1^· Afaf Zekri^1^ · Shafi Ullah Khan^2,3^ · Kannan R. R. Rengasamy ^4,5^ · Bader Y. Alhatlani^6^

Group of Computational and Medicinal Chemistry, LMCE Laboratory, University of Biskra, BP 145, 07000 Biskra Algeria

2 Inserm U1086 ANTICIPE (Interdisciplinary Research Unit for Cancer Prevention and Treatment), Universite de Caen Normandie, Normandie University, Caen, France

3 Comprehensive Cancer Center Francois Baclesse UNICANCER, 14076 Caen, France

4 Laboratory of Natural Products and Medicinal Chemistry (LNPMC), Department of Pharmacology, Saveetha Medical College and Hospitals, Saveetha Institute of Medical

and Technical Sciences (SIMATS), Thandalam, Chennai, India

5 Centre of Excellence for Pharmaceutical Sciences, North-West University, Potchefstroom 2520, South Africa

6 Unit of Scientific Research, Applied College Qassim University, 52571 Buraydah, Saudi Arabia

* Mebarka Ouassaf [nouassaf@univ-biskra.dz](mailto:nouassaf@univ-biskra.dz) * Bader Y. Alhatlan [balhatlani@qu.edu.sa](mailto:balhatlani@qu.edu.sa)


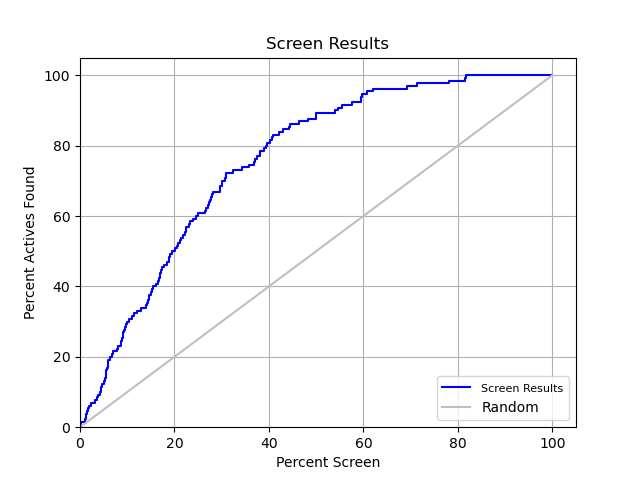


Figure S1 : ROC Curve Evaluating Docking Protocol Performance for JAK2 Inhibitor Identification


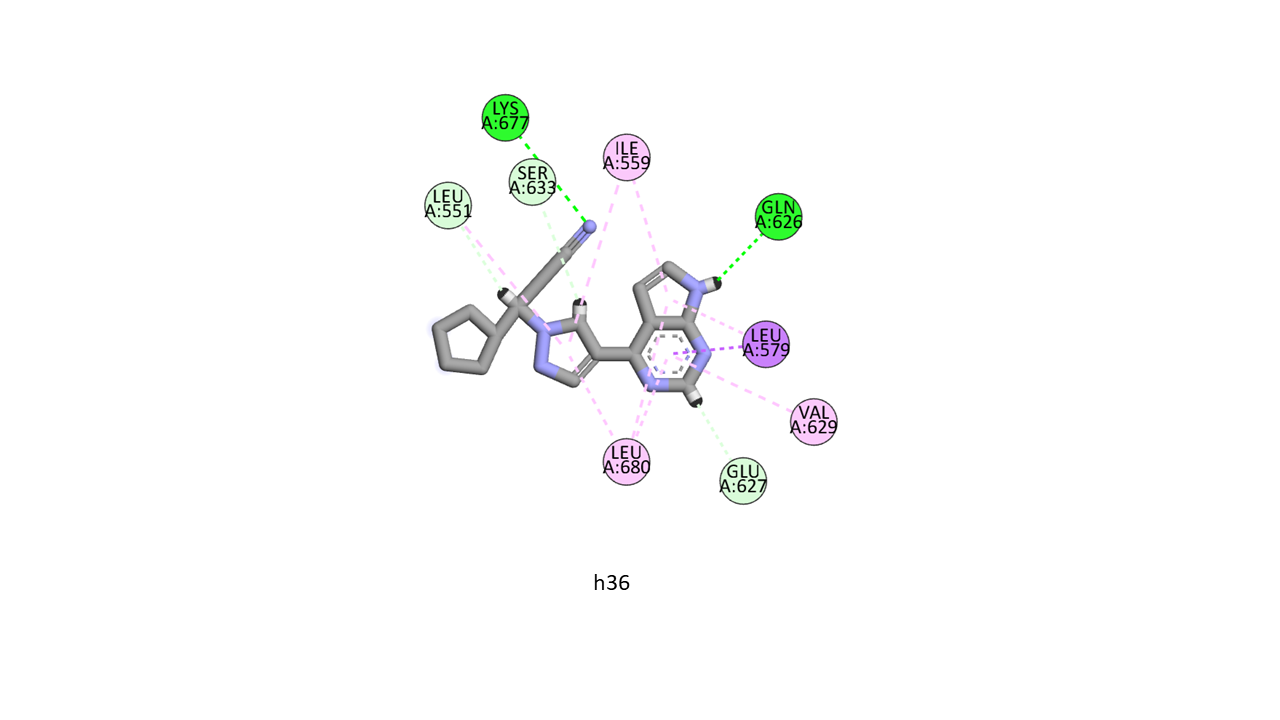


**Figure s2:** Molecular Binding Interactions of H36 with the JAK2 (JH2)


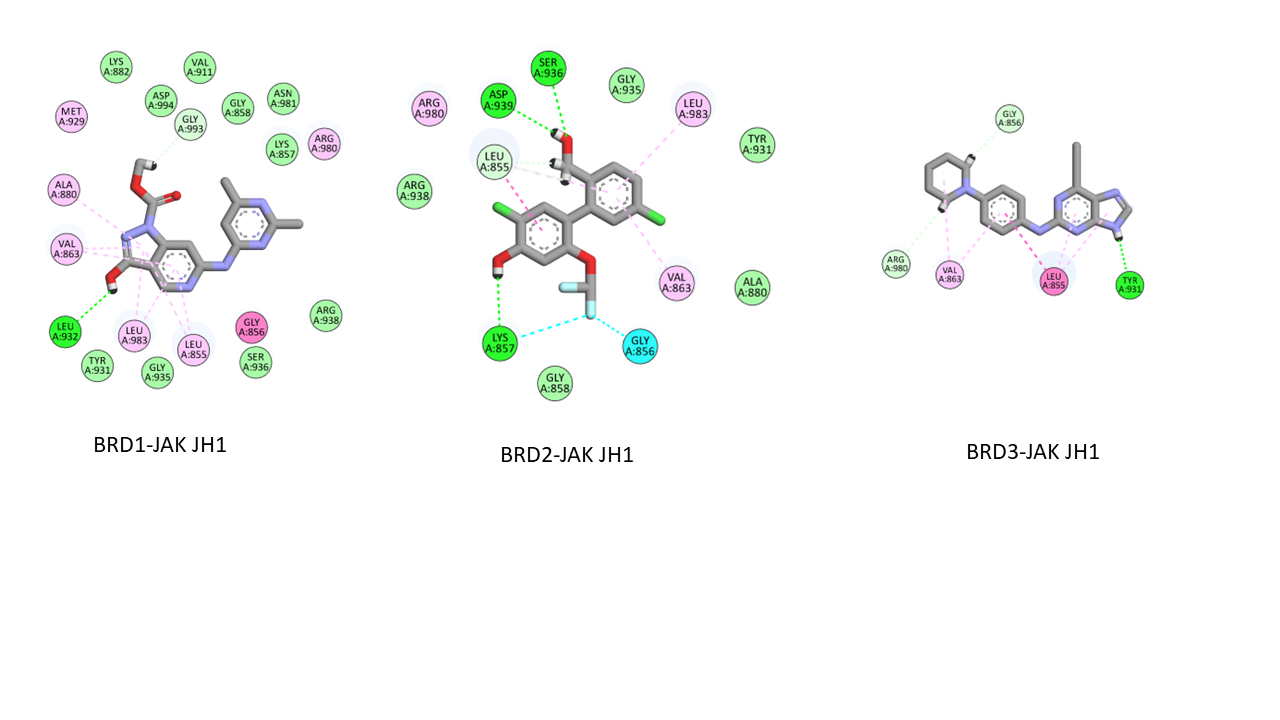


**Figure S3**/ Molecular Binding Interactions of Proposed Inhibitors with the JAK2 Catalytic Kinase Domain (JH1)

**Table S 1: SMILES Representation of 130 JAK2 Inhibitors Used as Parent Compounds for BREED-Based Hybridization**

| SMILES |
| --- |
| O=C(Nc1c[nH]nc1-c1cc(Cl)ccc1OC(F)F)c1cncc2ccoc12 |
| Nc1nn2cccnc2c1C(=O)Nc1c[nH]nc1-c1cc(Cl)ccc1OC(F)F |
| Cc1ccn2ncc(C(=O)Nc3c[nH]nc3-c3cc(Cl)ccc3OC(F)F)c2n1 |
| C#Cc1ccc2ncc(C(=O)Nc3c[nH]nc3-c3cc(Cl)ccc3OC(F)F)n2n1 |
| COc1ccc(Cl)cc1-c1nn(C)cc1NC(=O)c1cncc2[nH]nnc12 |
| O=C(Nc1c[nH]nc1-c1ccccc1OC(F)F)c1cnn2cccnc12 |
| O=C(Nc1c[nH]nc1-c1cc(I)ccc1OC(F)F)c1cnn2cccnc12 |
| O=C(Nc1c[nH]nc1-c1cc2ccccc2cc1OC(F)F)c1cnn2cccnc12 |
| CCc1cc(O)c(F)cc1-c1cc(NS(C)(=O)=O)c2cn[nH]c2c1 |
| CCS(=O)(=O)Nc1cc(-c2c(C)cc(O)cc2C)cc2[nH]ncc12 |
| CCS(=O)(=O)Nc1cc(-c2ccc(O)c(C)c2)cc2[nH]ncc12 |
| CCc1cc(O)c(F)cc1-c1cc(NS(=O)(=O)C(F)(F)F)c2cn[nH]c2c1 |
| CCc1cc(O)ccc1-c1ccc2c(-c3nc4c([nH]3)CN(S(C)(=O)=O)CC4)n[nH]c2c1 |
| CCS(=O)(=O)Nc1cc(-c2ccc(O)cc2C)cc2[nH]ncc12 |
| CCOC(=O)c1cc2c(=O)oc3cc(O)ccc3c2s1 |
| CNc1cc(Nc2cccn(-c3ccc(C)nc3)c2=O)nc2c(C(=O)N[C@H]3CC[C@@H]3OC)cnn12 |
| CCOC(=O)c1cc2c(=O)oc3cc(O)ccc3c2s1 |
| COc1ccc(Cl)cc1-c1nn(C)cc1NC(=O)c1cncc2cccnc12 |
| O=C(Nc1c[nH]nc1-c1cc(Cl)c(O)cc1OC(F)F)c1cnn2cccnc12 |
| O=C(Nc1c[nH]nc1-c1cc(Cl)ccc1OC1CC1)c1cnn2cccnc12 |
| O=C(Nc1c[nH]nc1-c1cc(Cl)ccc1OC(F)(F)F)c1cnn2cccnc12 |
| O=C(Nc1c[nH]nc1-c1cc(Cl)ccc1SC(F)F)c1cnn2cccnc12 |
| COc1cc(F)c(Cl)cc1-c1n[nH]cc1NC(=O)c1cnn2cccnc12 |
| COc1cc(O)c(Cl)cc1-c1n[nH]cc1NC(=O)c1cnn2cccnc12 |
| Cn1cc(NC(=O)c2cnn3cccnc23)c(-c2cc(Cl)ccc2C2CC2)n1 |
| CSc1ccc(Cl)cc1-c1nn(C)cc1NC(=O)c1cnn2cccnc12 |
| COc1cc2[nH]ncc2cc1-c1n[nH]cc1NC(=O)c1cnn2cccnc12 |
| COc1cc2ccccc2cc1-c1n[nH]cc1NC(=O)c1cnn2cccnc12 |
| CNc1cc(Nc2cccn(-c3ncccc3F)c2=O)nc2c(C(=O)N[C@@H]3CC[C@H]3OC)cnn12 |
| COc1ccc(Cl)c(F)c1-c1n[nH]cc1NC(=O)c1cnn2cccnc12 |
| Cc1ccc(OC(F)F)c(-c2n[nH]cc2NC(=O)c2cnn3cccnc23)c1 |
| CNc1cc(Nc2cccn(-c3ncccc3F)c2=O)nc2c(C(=O)N[C@@H]3CC[C@H]3OC)cnn12 |
| CNc1cc(Nc2cccn(-c3ccc(C)nc3)c2=O)nc2c(C(=O)N[C@H]3CC[C@@H]3OC)cnn12 |
| NC(=O)C1C2C=CC(C2)C1Nc1nc(Nc2cccc(S(N)(=O)=O)c2)nc2[nH]cnc12 |
| O=C(CCCCCCn1cc(Nc2ncc(Cl)c(Nc3ccc(Cl)cc3)n2)cn1)NO |
| CC(C)c1cc(O)ccc1-c1ccc2c(-c3nc4ccccc4[nH]3)n[nH]c2c1 |
| CCc1c(-c2ccc3c(-c4nc5ccccc5[nH]4)n[nH]c3c2)ccc(O)c1F |
| CCc1cc(O)ccc1-c1ccc2c(-c3nc4c([nH]3)CN(C)CC4)n[nH]c2c1 |
| Cn1cc(NC(=O)c2cnn3cccnc23)c(-c2cc(Cl)ccc2CCO)n1 |
| CNc1cc(Nc2cccn(-c3ncccc3F)c2=O)nc2c(C(=O)N[C@@H]3C[C@@H]3F)cnn12 |
| CC(C)Oc1ccc(Cl)cc1-c1nn(C)cc1NC(=O)c1cnn2cccnc12 |
| COc1ccc(Cl)cc1-c1nn(C)cc1NC(=O)c1cncc2c[nH]nc12 |
| O=C(Nc1c[nH]nc1-c1cc(F)ccc1OC(F)F)c1cnn2cccnc12 |
| CCc1ccc(OC(F)F)c(-c2n[nH]cc2NC(=O)c2cnn3cccnc23)c1 |
| O=C(Nc1c[nH]nc1-c1c(OC(F)F)ccc(Cl)c1F)c1cnn2cccnc12 |
| CCc1cc(O)c(F)cc1-c1cc(NS(=O)(=O)N(C)C)c2cn[nH]c2c1 |
| CCc1cc(O)c(F)cc1-c1cc(NS(=O)(=O)C2CCCC2)c2cn[nH]c2c1 |
| CCS(=O)(=O)Nc1cc(-c2ccc(O)c(F)c2)cc2[nH]ncc12 |
| CCc1cc(O)ccc1-c1ccc2c(C(=O)Nc3ccc(C)nc3)n[nH]c2c1 |
| CCOC(=O)c1cc2c(=O)oc3cc(O)ccc3c2s1 |
| CCc1cc(O)c(F)cc1-c1cc(NS(=O)(=O)CCOC)c2cn[nH]c2c1 |
| c1nc2c(NC3CC4CCC3C4)nc(Nc3ccc(N4CCCCC4)cc3)nc2[nH]1 |
| COc1cc(Nc2nc(NC3C4C=CC(C4)C3C(N)=O)c3nc[nH]c3n2)cc(OC)c1OC |
| CNc1cc(Nc2cccn(-c3ccccn3)c2=O)nc2c(C(=O)N[C@H]3CC[C@@H]3OC)cnn12 |
| CNc1cc(Nc2cccn(-c3ocnc3C)c2=O)nc2c(C(=O)N[C@@H]3CC[C@H]3OC)cnn12 |
| CCc1cc(O)c(F)cc1-c1ccc2c(-c3nc4c([nH]3)CCN(C(=O)c3cnc(N5CCCCC5)cn3)C4)n[nH]c2c1 |
| CCc1cc(O)ccc1-c1ccc2c(C(N)=O)n[nH]c2c1 |
| CCc1cc(O)ccc1-c1ccc2c(C(=O)NC)n[nH]c2c1 |
| CCc1cc(O)c(F)cc1-c1cc(NS(=O)(=O)CCN2CCCCC2)c2cn[nH]c2c1 |
| O=C(Nc1c[nH]nc1-c1cc(Cl)ccc1OC(F)F)c1cncc2scnc12 |
| O=C(Nc1c[nH]nc1-c1cc(Br)ccc1OC(F)F)c1cnn2cccnc12 |
| CCc1cc(O)c(F)cc1-c1cc(NS(=O)(=O)CCO)c2cn[nH]c2c1 |
| CS(=O)(=O)Nc1cc(-c2ccc(O)cc2)cc2[nH]ncc12 |
| CCS(=O)(=O)Nc1cc(-c2ccc(O)c(OC)c2)cc2[nH]ncc12 |
| CCc1cc(O)c(F)cc1-c1cc(N(C)S(C)(=O)=O)c2cn[nH]c2c1 |
| CCc1cc(O)c(F)cc1-c1ccc2c(-c3nc4c([nH]3)CCN(C(=O)c3ccc(F)cc3)C4)n[nH]c2c1 |
| Cc1nc(-c2cc(Cl)ccc2OC(F)F)c(NC(=O)c2cnn3cccnc23)[nH]1 |
| O=C(Nc1c[nH]nc1-c1cc(Cl)ccc1OC(F)F)c1cnn2cc(F)cnc12 |
| O=C(Nc1c[nH]nc1-c1cc(Cl)ccc1OC(F)F)c1cncc2c[nH]nc12 |
| Cn1cc(NC(=O)c2cnn3cccnc23)c(-c2cc(Cl)ccc2O)n1 |
| COc1ccc(Cl)cc1-c1n[nH]cc1NC(=O)c1cnn2cccnc12 |
| C[C@@H](O)c1nc2cnc3[nH]ccc3c2n1[C@H]1CCCOC1 |
| COc1c(F)cc(Cl)cc1-c1n[nH]cc1NC(=O)c1cnn2cccnc12 |
| Cc1cc(NC(=O)c2cnn3cccnc23)n(-c2cccc(Cl)c2)n1 |
| Cn1ncc(NC(=O)c2cnn3cccnc23)c1-c1cc(Cl)ccc1Cl |
| Cn1cc(NC(=O)c2cnn3cccnc23)c(-c2cc(Cl)ccc2CO)n1 |
| CCOc1ccc(Cl)cc1-c1n[nH]cc1NC(=O)c1cnn2cccnc12 |
| N#Cc1ccc(OC(F)F)c(-c2n[nH]cc2NC(=O)c2cnn3cccnc23)c1 |
| CCc1cc(O)ccc1-c1ccc2c(C(=O)N(C)C)n[nH]c2c1 |
| CCc1cc(O)c(F)cc1-c1ccc2c(-c3nc4c([nH]3)CN(Cc3ccccc3)CC4)n[nH]c2c1 |
| C[C@@H]1CCN(C(=O)N2CC[C@@H](S(=O)(=O)c3cccc(O)c3)C2)C[C@@H]1N(C)c1ncnc2[nH]ccc12 |
| CCc1cc(O)c(F)cc1-c1ccc2c(-c3nc4ccccc4[nH]3)n[nH]c2c1 |
| CCc1cc(O)c(F)cc1-c1cc(NS(=O)(=O)CC2CCC2)c2cn[nH]c2c1 |
| CCCc1cc(O)ccc1-c1ccc2c(-c3nc4ccccc4[nH]3)n[nH]c2c1 |
| CCc1cc(O)c(F)cc1-c1cc(NS(=O)(=O)CC)c2cn[nH]c2c1 |
| CCc1cc(O)c(F)cc1-c1cc(NS(=O)(=O)c2ccccc2Cl)c2cn[nH]c2c1 |
| Oc1cccc(-c2ccc3c(-c4nc5ccccc5[nH]4)n[nH]c3c2)c1 |
| CCn1c(C(=O)N(C2CC2)C2CC2)cc2c3c(ncn3C)c(Nc3cc(C)n(C)n3)nc21 |
| CCc1cc(O)c(F)cc1-c1cc(NS(=O)(=O)c2cccnc2)c2cn[nH]c2c1 |
| CCc1cc(O)c(F)cc1-c1cc(NS(=O)(=O)c2ccccn2)c2cn[nH]c2c1 |
| CCS(=O)(=O)Nc1cc(-c2ccc(O)c(Cl)c2)cc2[nH]ncc12 |
| N#CC[C@H](C1CCCC1)n1cc(-c2ncnc3[nH]ccc23)cn1 |
| COc1ccc(Cl)cc1-c1nn(C)cc1NC(=O)c1cnn2cccnc12 |
| Cn1cc(NC(=O)c2cnn3cccnc23)c(-c2cc(Cl)ccc2OC(F)F)n1 |
| CCc1cc(O)c(F)cc1-c1cc(NS(=O)(=O)C2CC2)c2cn[nH]c2c1 |
| Cn1cc(NC(=O)c2cnn3cccnc23)c(-c2cccc(Cl)c2)n1 |
| O=C(Nc1c[nH]nc1-c1cc(Cl)ccc1OC(F)F)c1cnc2[nH]ccc2n1 |
| COc1ccc(Cl)cc1-c1nn(C)cc1NC(=O)c1cncc2[nH]cnc12 |
| C[C@@H]1CCN(C(=O)N2CCCC2)C[C@@H]1N(C)c1ncnc2[nH]ccc12 |
| CCc1cc(O)ccc1-c1ccc2c(-c3nc4ccccc4[nH]3)n[nH]c2c1 |
| CCc1cc(O)ccc1-c1ccc2c(-c3ncc[nH]3)n[nH]c2c1 |
| CCc1cc(O)ccc1-c1ccc2c(-c3nc4c([nH]3)CN(C(C)=O)CC4)n[nH]c2c1 |
| COc1cccc(-n2[nH]c(=O)c3cnc(Nc4cc(C)nc(C)n4)cc32)c1F |
| CCc1cc(O)c(F)cc1-c1cc(NS(=O)(=O)CCC(=O)OC)c2cn[nH]c2c1 |
| CCS(=O)(=O)Nc1cc(-c2ccc(O)cc2Cl)cc2[nH]ncc12 |
| CCc1cc(O)ccc1-c1cc(NS(=O)(=O)CC)c2cn[nH]c2c1 |
| CCS(=O)(=O)Nc1cc(-c2ccc(O)cc2)cc2[nH]ncc12 |
| O=C(Nc1c[nH]nc1-c1cc(Cl)ccc1OC(F)F)c1cnn2cccnc12 |
| O=C(Nc1c[nH]nc1-c1cc(Cl)ccc1OC(F)F)c1cnc2cccnn12 |
| COc1ccc(Cl)cc1-c1nn(C)cc1NC(=O)c1cncc2cn(C)nc12 |
| CCc1cc(O)c(F)cc1-c1cc(NS(=O)(=O)CCN2CCN(C)CC2)c2cn[nH]c2c1 |
| CCc1cc(O)c(F)cc1-c1cc(NS(=O)(=O)C(C)C)c2cn[nH]c2c1 |
| CNc1cc(Nc2cccn(-c3ncccc3F)c2=O)nc2c(C(=O)N[C@@H]3C[C@@H]3F)cnn12 |
| CCc1cc(O)c(F)cc1-c1cc(NP(=O)(CC)CC)c2cn[nH]c2c1 |
| CC(C)Oc1cccc(Nc2nc(NC3C4C=CC(C4)C3C(N)=O)c3nc[nH]c3n2)c1 |
| CNc1cc(Nc2cccn(-c3ccccn3)c2=O)nc2c(C(=O)N[C@H]3CC[C@@H]3OC)cnn12 |
| CNc1cc(Nc2cccn(-c3ocnc3C)c2=O)nc2c(C(=O)N[C@@H]3CC[C@H]3OC)cnn12 |
| N#Cc1ccc(-n2[nH]c(=O)c3cnc(Nc4ccc(F)cn4)cc32)cc1Cl |
| O=C(Nc1c[nH]nc1-c1cc(Cl)ccc1OC(F)F)c1snc2cccnc12 |
| O=C(Nc1c[nH]nc1-c1cc2cn[nH]c2cc1OC(F)F)c1cnn2cccnc12 |
| NC(=O)[C@@H]1[C@H](Nc2nc(Nc3cccc(S(N)(=O)=O)c3)nc3[nH]cnc23)[C@H]2C=C[C@@H]1C2 |
| CC(C)(/C=C(\C#N)C(=O)N1CCC[C@H](n2nc(-c3ccc(Oc4ccccc4)cc3F)c3c(N)ncnc32)C1)N1CCN(C2COC2)CC1 |
| COc1ccc(Cl)cc1-c1nc(C)sc1NC(=O)c1cnn2cccnc12 |
| N#CCC1CCN(c2nc(Nc3ccc(N4CCC(O)CC4)cc3)c3c(=O)[nH]ncc3n2)CC1 |
| Cc1cc(Nc2nccc3nc(-c4c(Cl)cc(C#N)cc4Cl)[nH]c23)ncn1 |
| C[C@@H]1CCN(C(=O)CC#N)C[C@@H]1N(C)c1ncnc2[nH]ccc12 |
| COc1cc(O)ccc1-c1ccc2c(-c3nc4ccccc4[nH]3)n[nH]c2c1 |
| CC[C@@H]1CN(C(=O)NCC(F)(F)F)C[C@@H]1c1cnc2cnc3[nH]ccc3n12 |
| Cn1cc(NC(=O)c2cnn3cccnc23)c(-c2cc(Cl)ccc2Cl)n1 |
| Cc1cnc(Nc2ccc(OCCN3CCCC3)cc2)nc1Nc1cccc(S(=O)(=O)NC(C)(C)C)c1 |

**Table s2:** Summary of the Enrichment Validation for the Docking Protocol

| **Parameter** | **Value** |
| --- | --- |
| ROC (Receiver Operating Characteristic) | 0.79 |
| Area under accumulation curve | 0.76 |
| BEDROC (α=160.9) | 0.351 |
| BEDROC (α=20.0) | 0.343 |
| BEDROC (α=8.0) | 0.455 |
| RIE (Robust Initial Enhancement) | 2.69 |
| EF (1%) | 1.6 |
| EF (2%) | 3.0 |
| EF (5%) | 2.5 |
| EF (10%) | 3.0 |
| EF (20%) | 2.5 |
